# Nef Suppresses LINE-1 Retrotransposition through Two Distinct Mechanisms

**DOI:** 10.1128/jvi.01148-22

**Published:** 2022-10-05

**Authors:** Yu Wang, Ke Zhao, Yifei Zhao, Zihan Zhao, Shaohua Wang, Juan Du

**Affiliations:** a Center of Infectious Diseases and Pathogen Biology, First Hospital of Jilin University, Changchun, Jilin, China; b Institute of Virology and AIDS Research, First Hospital of Jilin University, Changchun, Jilin, China; c Key Laboratory of Organ Regeneration & Transplantation of the Ministry of Education, First Hospital of Jilin University, Changchun, Jilin, China; Icahn School of Medicine at Mount Sinai

**Keywords:** Nef, human immunodeficiency virus, long interspersed element type 1, promoter regulation, interaction interruption, interferon

## Abstract

Long interspersed element type 1 (LINE-1) is the only known type of retroelement that can replicate autonomously, and its retrotransposition activity can trigger interferon (IFN) production. IFN production suppresses the infectivity of exogenous viruses, such as human immunodeficiency virus (HIV). As a counteraction, HIV has been reported to use multiple proteins and mechanisms to suppress LINE-1 replication. However, the mechanisms of HIV-mediated LINE-1 regulation are not fully understood. In this study, we discovered that Nef protein, which is expressed by HIV and is important for HIV pathogenesis, inhibits LINE-1 retrotransposition. Two distinct mechanisms have been uncovered for Nef-induced LINE-1 suppression. Without direct interaction with LINE-1 DNA, Nef potently inhibits the promoter activity of the LINE-1 5′-untranslated region (5′-UTR) and reduces the expression levels of LINE-1 RNA and proteins. Alternatively, although Nef does not bind to the LINE-1 open reading frame 1 protein (ORF1p) or LINE-1 RNA, it significantly compromises the ORF1p-LINE-1 RNA interaction, which is essential for LINE-1 retrotransposition. Both mechanisms can be suppressed by the G2A mutation, which abolishes myristoylation of Nef, suggesting that membrane attachment is essential for Nef to suppress LINE-1. Consequently, through LINE-1 inhibition, Nef downregulates IFN production in host cells. Therefore, our data revealed that Nef is a potent LINE-1 suppressor and an effective innate immune regulator, which not only provides new information on the intricate interaction between HIV, LINE-1, and IFN signaling systems but also strengthens the importance of Nef in HIV infection and highlights the potential of designing novel Nef-targeting anti-HIV drugs.

**IMPORTANCE** Human immunodeficiency viruses are pathogens of AIDS that were first discovered almost 40 years ago and continue to threaten human lives to date. While currently used anti-HIV drugs are sufficient to suppress viral loads in HIV-infected patients, both drug-resistant HIV strains and adverse side effects triggered by the long-term use of these drugs highlight the need to develop novel anti-HIV drugs targeting different viral proteins and/or different steps in viral replication. To achieve this, more information is required regarding HIV pathogenesis and especially its impact on cellular activities in host cells. In this study, we discovered that the Nef protein expressed by HIV potently inhibits LINE-1 retrotransposition. During our attempt to determine the mechanism of Nef-mediated LINE-1 suppression, two additional functions of Nef were uncovered. Nef effectively repressed the promoter activity of LINE-1 5′-UTR and destabilized the interaction between ORF1p and LINE-1 RNA. Consequently, Nef not only compromises LINE-1 replication but also reduces LINE-1-triggered IFN production. The reduction in IFN production, in theory, promotes HIV infectivity. Together with its previously known functions, these findings indicate that Nef is a potential target for the development of novel anti-HIV drugs. Notably, the G2 residue, which has been reported to be essential for most Nef functions, was found to be critical in the regulation of innate immune activation by Nef, suggesting that compromising myristoylation or membrane attachment of Nef may be a good strategy for the inhibition of HIV infection.

## INTRODUCTION

In humans, long interspersed element type 1 (LINE-1 or L1) is the only type of endogenous retroelement that can replicate autonomously (1, 2). A typical LINE-1 fragment is 6 kb in length, containing two open reading frames (named *orf1* and *orf2*, respectively) flanked by 5′- and 3′-untranslated regions (UTRs). The expressed proteins ORF1p and ORF2p are essential for LINE-1 retrotransposition. They interact with LINE-1 RNA and trigger the assembly of LINE-1 ribonucleoprotein particles (RNPs), which are the fundamental units for LINE-1 replication (3, 4). In addition, ORF2p possesses both endonuclease and reverse transcriptase activities (5, 6), which not only facilitate LINE-1 retrotransposition but also support the replication of other nonautonomous yet active retroelements, such as Alu and SVA (7, 8). Therefore, due to the vast copy numbers of all these retroelements, LINE-1 is responsible for the generation of >30% of the human genomic DNA (9, 10). Consistently, previous studies have revealed a link between LINE-1 activity and genome evolution, including epigenetic regulation, 5′ and 3′ transduction, exon skipping, transcription termination, and gene breaking and silencing (11–15).

Endogenous retroelements and exogenous retroviruses resemble each other in several ways; for example, both replicate via reverse transcription and genome integration. Many host restriction factors are known to suppress re plication of both HIV-1 and LINE-1. Surprisingly, detailed analyses indicated that some of these factors inhibit HIV-1 replication and LINE-1 retrotransposition through different mechanisms. For example, deoxynucleoside triphosphate (dNTP) depletion is essential for SAMHD1 to repress HIV-1 (16), whereas ORF2p degradation is the major mechanism by which SAMHD1 suppresses LINE-1 (17, 18). TREX1 downregulates the DNA levels of the reverse-transcribed HIV genome with its exonuclease activity (19) but reduces the protein levels of ORF1p through protease-mediated proteolysis (20). APOBEC3 proteins bind and introduce guanosine-to-adenosine mutations to HIV DNA (21), yet they interact with and compromise the function of LINE-1 RNPs (22). We recently proposed possible reasons for such phenomena (23), wherein the main cause may be the expected outcome of suppression. The goal of suppressing exogenous HIV is to prevent the spread of the virus inside the body. Accordingly, host restriction factors can act on all steps of the HIV replication process. The purpose of suppressing endogenous LINE-1 is to protect the integrity of the host genome and maintain a certain level of innate immune activation in the cell. Indeed, it has long been known that LINE-1 retrotransposition induces nicking and destabilizes genomic DNA (13, 24), while it has been recently revealed that LINE-1 replication activates innate immune regulation through both DNA- and RNA-sensing pathways to support host antiviral mechanisms and, if unregulated, causes autoimmune diseases (25, 26). Notably, LINE-1 RNPs are the fundamental units for all consequences of LINE-1 retrotransposition; it is therefore reasonable that restrictive factors suppress LINE-1 activity through mechanisms limiting LINE-1 RNP formation/function.

Intriguingly, in contrast to the fact that some host anti-HIV factors are LINE-1 inhibitors, some HIV proteins that support viral replication still suppress LINE-1 activity. For instance, HIV Vpr can induce cell cycle arrest, which enhances viral replication (27, 28) while repressing LINE-1 retrotransposition by interacting with and compromising ORF2p (29). HIV Vpu induces the depletion of the host restriction factor BST2 and promotes the release of newly assembled HIV virions (30, 31). Although BST2 is a potent LINE-1 suppressor (32), Vpu by itself also inhibits LINE-1 effectively (33). This is puzzling at first glance because similar effects are expected owing to the various similarities shared by retroviruses and retroelements. There are at least two reasons for these findings. As mentioned earlier, the replication process of LINE-1 triggers innate immune activation (25, 26, 34), and we have observed that the endogenous interferon (IFN) levels maintained by LINE-1 contribute to host defense against HIV infection (26). In addition, the reverse-transcribed HIV DNA has to be inserted into the host genome to initiate the synthesis of new virions, which may be interrupted by LINE-1-induced nicking or integration. Thus, to increase viral infection/replication, HIV may suppress LINE-1 activity.

In this study, we found that the Nef protein from HIV-1 potently suppresses exogenous LINE-1 activity *in vitro*. Further research on the mechanism revealed that Nef inhibits the promoter activity of the LINE-1 5′-UTR, reducing the expression of LINE-1 RNA and both LINE-1 proteins. In addition, Nef was found to compromise the assembly of LINE-1 RNPs by indirectly inhibiting the interaction between ORF1p and LINE-1 RNA. Surprisingly, the G2A mutation that blocks myristoylation reduces Nef’s potency in both mechanisms, indicating that membrane attachment is important for Nef-mediated LINE-1 regulation. At the same time, Nef also prevents LINE-1-induced IFN activation, which may contribute to the effectiveness of HIV replication.

## RESULTS

### Nef acts as a potent LINE-1 suppressor in LINE-1 retrotransposition assays.

**FIG 1:**
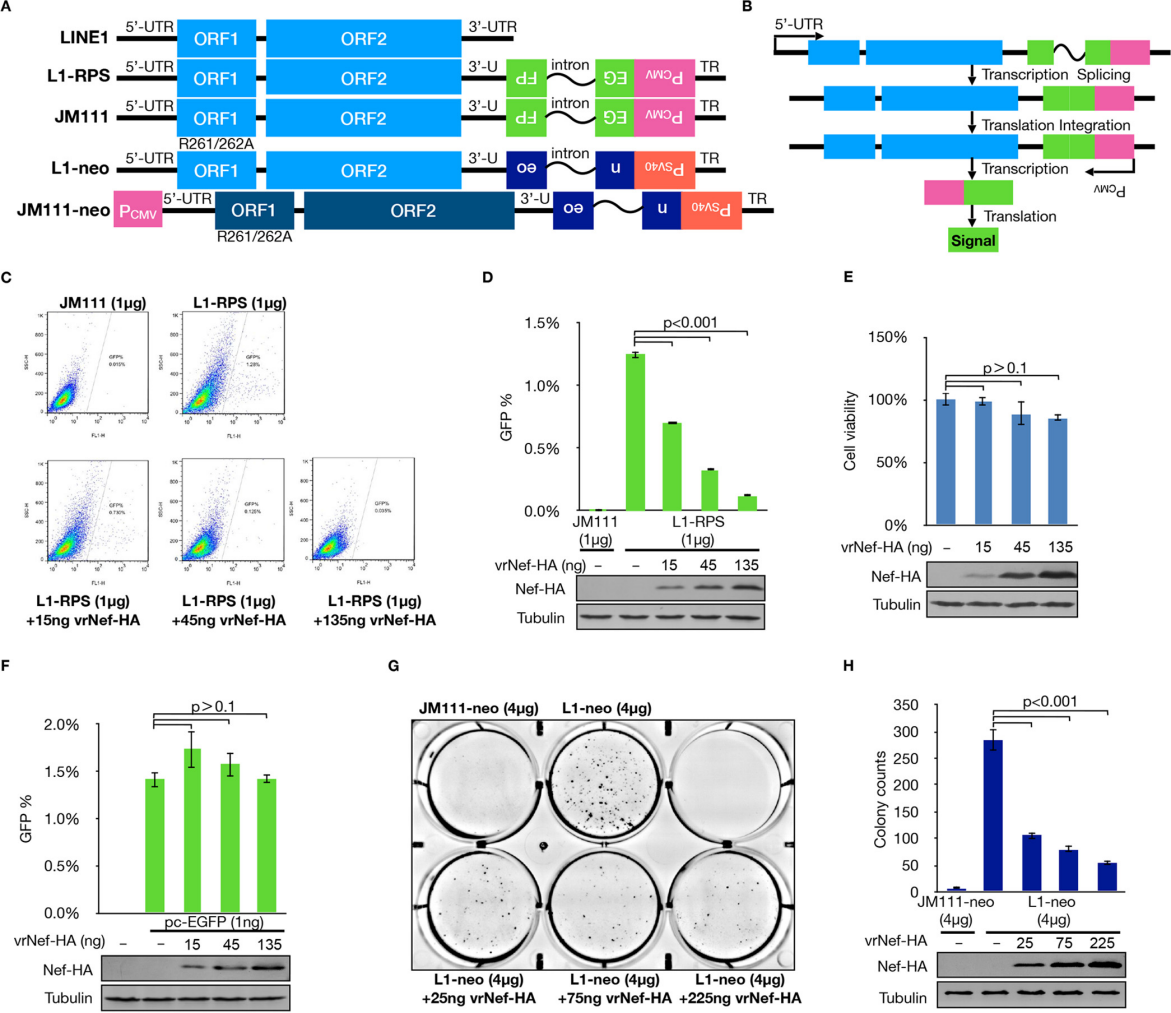
HIV Nef effectively suppresses LINE-1 retrotransposition. (A) Schematic of retrotransposition-competent LINE-1 plasmids L1-RPS and L1-neo and their negative controls JM111 and JM111-neo. L1-RPS is based on L1_RP_ and uses EGFP as the reporter to indicate a successful retrotransposition event. Derived from L1-RPS, JM111 contains two missense mutations in the ORF1p-coding region and thus becomes incompetent in retrotransposition. Similar to L1-RPS, L1-neo is also based on L1_RP_ but uses neomycin (G418) resistance as the reporter for retrotransposition. In contrast, JM111-neo is based on L1.2 instead of L1_RP_; it also contains a CMV promoter at the 5′ end of the LINE-1 sequence. However, it shares the same mutation in ORF1p-coding region as JM111 (EGFP based) and is also incompetent in retrotransposition; thus, it was used as the negative control in neomycin resistance-based LINE-1 assay. (B) Schematic of the rationale of signal-based LINE-1 retrotransposition assay. For example, the antisense *EGFP* gene in L1-RPS could not be translated directly, as it is interrupted by a sense intron. EGFP could only be expressed with when L1-RPS is transcribed, spliced (to remove the intron), reverse transcribed, and integrated into the host genome. The R261A/R262A mutation in ORF1p abolishes the retrotransposition potency of JM111, which was used as a negative control. A similar scenario also applies to L1-neo and JM111-neo, where neomycin resistance is considered the positive signal. (C) Representative images for the flow cytometry data shown in panel D. (D) Flow cytometry data suggesting that Nef suppresses L1-RPS retrotransposition in a dose-dependent manner in HEK293T cells. (E) CCK-8-based cell viability test results indicating that exogenous Nef expression barely triggers cytotoxicity in HEK293T cells. (F) Flow cytometry data showing that Nef does not affect EGFP expression driven by a CMV promoter. (G) Representative image showing Giemsa-stained cell clones in LINE-1 retrotransposition assays based on neomycin resistance. (H) Bar chart showing that Nef potently reduces L1-neo retrotransposition in a dose-dependent manner in HeLa-HA cells.

We first extracted the *nef* gene from an HIV-1 NL4-3 proviral DNA vector and inserted it into VR1012, a eukaryotic expression vector (35). NL4-3 is a laboratory-generated HIV recombinant strain from two isolated viruses, NY5 and LAV (36). NL4-3 and its encoded proteins are widely used as representatives in HIV-associated tests, such as to examine viral protein function(s) or viral infections. To determine whether Nef could suppress LINE-1 activity, we used a widely used enhanced green fluorescent protein (EGFP)-based LINE-1 retrotransposition assay, which includes the use of LINE-1 constructs 99 PUR RPS EGFP (L1-RPS) and 99 PUR JM111 EGFP (JM111) (Fig. 1A). In L1-RPS, the 3′ UTR of the LINE-1 fragment was interrupted by an antisense EGFP-expressing cassette, while the *EGFP* gene was further subdivided by a sense intron. Accordingly, the EGFP signal can only be detected when the LINE-1 transcript is spliced and reverse transcribed, its cDNA is inserted into the host genome, and the EGFP reporter gene is expressed from its own cytomegalovirus (CMV) promoter (Fig. 1B) (37). JM111 contains two missense mutations and, thus, is retrotransposition incompetent and used as a negative control (Fig. 1A). HEK293T cells were then cotransfected with L1-RPS and a Nef-expressing vector (or VR1012 as a control) and subjected to flow cytometry at 96 h posttransfection. The results indicated that exogenous expression of Nef significantly reduced the replication activity of the retrotransposition-competent L1-RPS vector in HEK293T cells (Fig. 1C and D). Cell counting kit-8 (CCK-8)-based tests suggested that the expression levels of Nef in the previously mentioned LINE-1 retrotransposition assays did not trigger cytotoxicity in HEK293T cells (Fig. 1E). Furthermore, Nef did not compromise EGFP expression driven by the CMV promoter or the stability of EGFP (Fig. 1F). These data indicated that the observed suppressive effect in Fig. 1C and D was due to the action of Nef on LINE-1 rather than on the reporter system or cell viability.

To further confirm the specific action of Nef on LINE-1, we introduced another widely used LINE-1 assay. L1-neo has a structure similar to that of L1-RPS, with the *EGFP* gene replaced by *mneoI*, the expression of which is driven by a simian virus 40 (SV40) promoter. Accordingly, successful retrotransposition of L1-neo allows cells to survive and form colonies under neomycin selection (Fig. 1A and B) (38). HeLa-hemagglutinin (HA), instead of HEK293T, cells were used in this assay because they form better colonies for detection. Similar to the results of the L1-RPS-based LINE-1 assay, the presence of Nef significantly suppressed the retrotransposition activity of L1-neo in HeLa-HA cells (Fig. 1G and H). These results suggest that NL4-3 Nef functions as a potent LINE-1 suppressor in LINE-1 retrotransposition assays.

### Nef reduces LINE-1 expression through 5′-UTR regulation.

**FIG 2:**
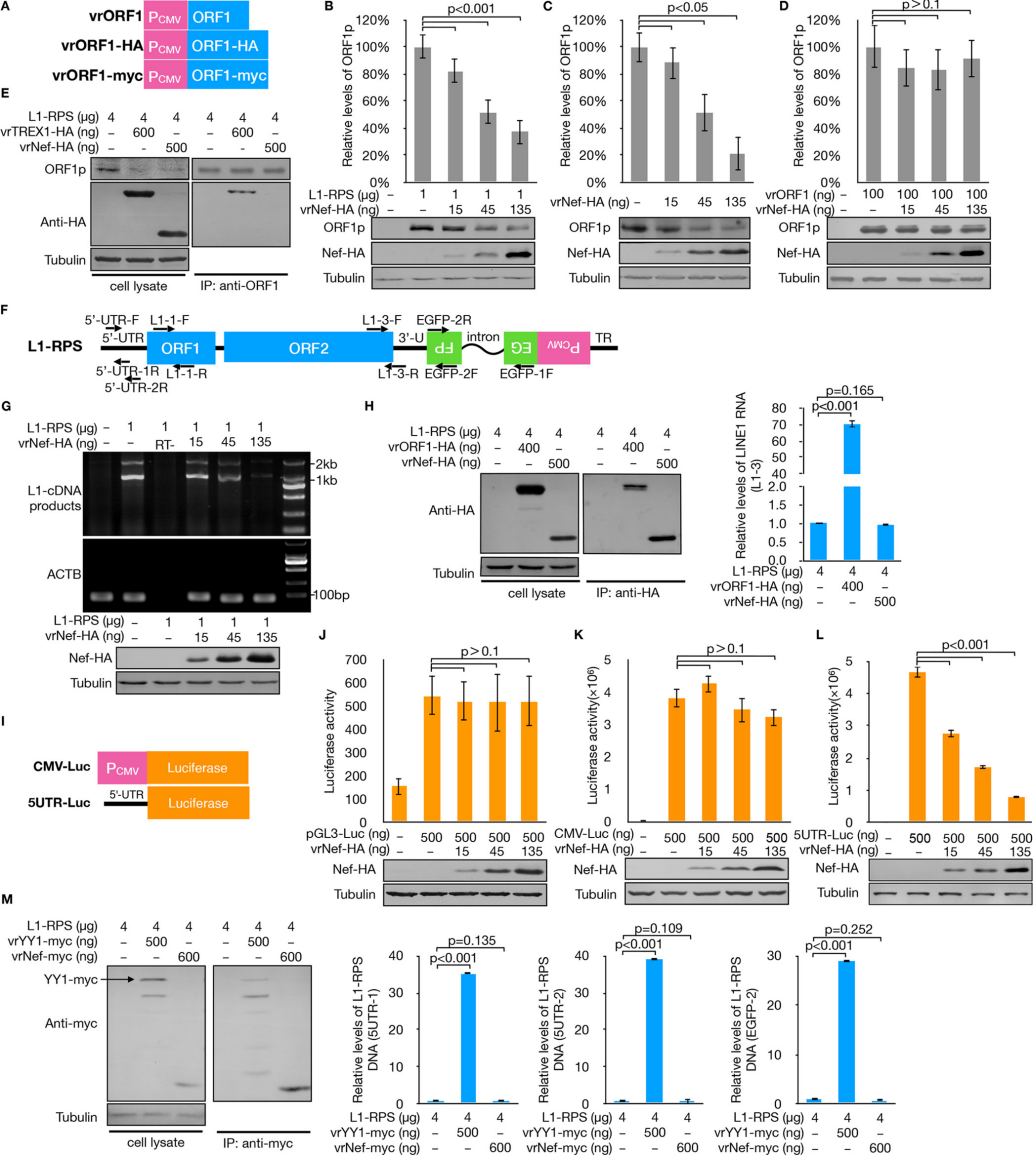
Nef suppresses LINE-1 retrotransposition by reducing the promoter activity of the LINE-1 5′-UTR. (A) Schematics of ORF1p-expressing vectors used in this study. (B to D) Bar charts showing relative levels of ORF1p expressed from L1-RPS (B), endogenously (C), or from vrORF1 (D) in the presence of exogenous Nef protein in HEK293T cells. The data were generated based on results from three independent experiments. Western blotting results are also shown. (E) Co-IP test results indicating that Nef does not interact with ORF1p. TREX1 (with an HA tag at its C terminus) was used as a positive control for ORF1p interaction. The loading amounts of the co-IP samples were adjusted based on eluted levels of ORF1p. (F) Schematic showing all primer target sites on L1-RPS for qRT-PCR tests. Notably, L1-3F and EGFP-1F were also used in PCR assays detecting levels of full-length L1-RPS RNA. (G) Images of electrophoresis gel showing that Nef efficiently downregulates the RNA levels transcribed from L1-RPS. The 2-kb bands in tested samples are unspliced L1-RPS RNA, whereas the 1.4-kb bands are spliced. Fragments of *ACTB* mRNA were also amplified and used as loading control. (H) qRT-PCR results indicating that Nef does not interact with L1-RPS RNA. L1-RPS (4 μg) and vrORF1-HA (400 ng) or vrNef-HA (500 ng) were cotransfected into HEK293T cells seeded on a 6-well plate. Transfected cells were subjected to co-IP tests at 48 h posttransfection, followed by qRT-PCR assay to detect interacted L1-RPS RNA. Western blotting results on the left show the ORF1p and Nef protein levels in the eluants. (I) Schematic showing luciferase-expressing cassettes in CMV-Luc and 5UTR-Luc vectors. (J) Luciferase assay results showing spontaneous expression levels of luciferase from pGL3-Luc, which is not affected by exogenous Nef expression. (K) Luciferase assay results indicating that Nef does not affect CMV promoter activity. (L) Luciferase assay results indicating that Nef suppresses the promoter activity of the LINE-1 5′-UTR. (M) qRT-PCR results indicating that Nef does not interact with L1-RPS plasmid DNA. HEK293T cells seeded on a 6-well plate were cotransfected with L1-RPS (4 μg) and Nef (600 ng) or a YY1 (500 ng)-expressing vector. Transfected cells were subjected to co-IP at 48 h posttransfection, followed by DNA extraction and qRT-PCR to detect interacted L1-RPS DNA (with primer pairs targeting the 5′-UTR or the second *EGFP* gene fragment of L1-RPS). Western blotting results on the left show the YY1 and Nef protein levels in the eluants. YY1 was included as a positive control for L1-RPS interaction.

Known LINE-1 suppressors mostly target LINE-1 RNA or one of two LINE-1 proteins (see reference 39 for more details). To investigate the mechanism of Nef-mediated LINE-1 suppression, we examined whether Nef affects the expression of LINE-1 proteins. Additional ORF1p-expressing vectors were also introduced (Fig. 2A). We first tested whether Nef could affect ORF1p levels expressed by the LINE-1 fragment, and we used HEK293T cells transfected with L1-RPS and Nef-expressing vector. As shown in Fig. 2B, the levels of ORF1p expressed by L1-RPS decreased in the presence of exogenous Nef in a dose-dependent manner. A similar reduction was observed for endogenous ORF1p levels in HEK293T cells expressing exogenous Nef (Fig. 2C). However, Nef could no longer reduce ORF1p expression in vrORF1 (Fig. 2D), which only expressed an untagged ORF1p and contained no other LINE-1 fragment (Fig. 2A). These findings suggest that Nef-induced protein degradation (e.g., of SERINC proteins [40, 41]) may not apply to Nef-mediated ORF1p reduction. Consistent with this, subsequent coimmunoprecipitation (co-IP) tests indicated that Nef did not interact with ORF1p, despite the previously reported TREX1-ORF1p interaction being readily detected in parallel tests (Fig. 2E) (20). Therefore, Nef did not decrease ORF1p levels via a posttranslational mechanism.

The fact that Nef reduced ORF1p without targeting it led to the assumption that Nef may affect the generation or stability of LINE-1 RNA. Previously, we developed an assay to detect the levels of full-length LINE-1 RNA using an exogenous LINE-1 plasmid (Fig. 2F) (20). Similarly, in this study, RNA was extracted from HEK293T cells transfected with L1-RPS 48 h posttransfection and reverse transcribed. The synthesized LINE-1 cDNA was then subjected to PCR with primers L1-3F and EGFP-1F. L1-3F targets the 3′ end of the LINE-1 fragment, while EGFP-1F targets the 5′ end of the *EGFP* gene (Fig. 2F), which ensures that the amplicon is derived from L1-RPS transcripts rather than transcripts directly from the EGFP cassette or the numerous LINE-1 copies in the human genome. As shown in Fig. 2G, exogenous Nef potently decreased the levels of exogenous LINE-1 RNA. We performed co-IP tests to retrieve Nef interactants, where exogenous ORF1p was introduced as a positive control. However, subsequent quantitative real-time reverse transcription-PCR (qRT-PCR) tests indicated that, unlike ORF1p, Nef did not interact with LINE-1 RNA (Fig. 2H), ruling out the possibility of Nef destabilizing LINE-1 RNA, at least not directly.

The 5′-UTR functions as a promoter of LINE-1 transcription. We recently confirmed that suppressing the 5′-UTR contributes to BST2-mediated LINE-1 inhibition (32). Thus, the data described might indicate that Nef suppresses the generation of LINE-1 RNA through 5′-UTR repression. We introduced a firefly luciferase-based assay to examine the promoter activity of the LINE-1 5′-UTR in the presence of the Nef protein (Fig. 2I) (32). This system was based on the pGL3-basic vector, which can spontaneously express luciferase even without the presence of a proper promoter (Fig. 2J). Notably, cotransfection with the Nef-expressing vector did not affect luciferase levels expressed by pGL3-basic (Fig. 2J), which confirmed that Nef did not trigger cytotoxicity in HEK293T cells. This also suggested that Nef did not compromise the stability of the luciferase protein. Similar tests indicated that Nef did not affect luciferase expression driven by the CMV promoter (Fig. 2K), which is consistent with our previous observation that Nef did not affect EGFP expression driven by the CMV promoter (Fig. 1F). Nef significantly compromised the expression of luciferase driven by the LINE-1 5′-UTR (Fig. 2L). We then determined whether Nef regulates the 5′-UTR through direct DNA interaction, and HEK293T cells were transfected with both L1-RPS and Nef-expressing vectors. YY1, which has been reported to regulate LINE-1 transcription, was introduced as a positive control for 5′-UTR binding (42, 43). However, co-IP and subsequent qRT-PCR tests indicated that as the YY1-L1-RPS interaction was readily detected (likely through YY1 binding LINE-1 5′-UTR), Nef did not interact with the LINE-1 5′-UTR or any other part of the L1-RPS plasmid (Fig. 2M). It therefore also ruled out the possibility of Nef blocking transcription by binding to the non-5′-UTR of LINE-1 DNA. In total, these data suggest that Nef suppressed LINE-1 activity in the LINE-1 retrotransposition assay by sabotaging the promoter activity of the LINE-1 5′-UTR indirectly and compromising the subsequent synthesis of LINE-1 RNA and proteins.

### Myristoylation is essential for the suppression of LINE-1 by Nef.

**FIG 3:**
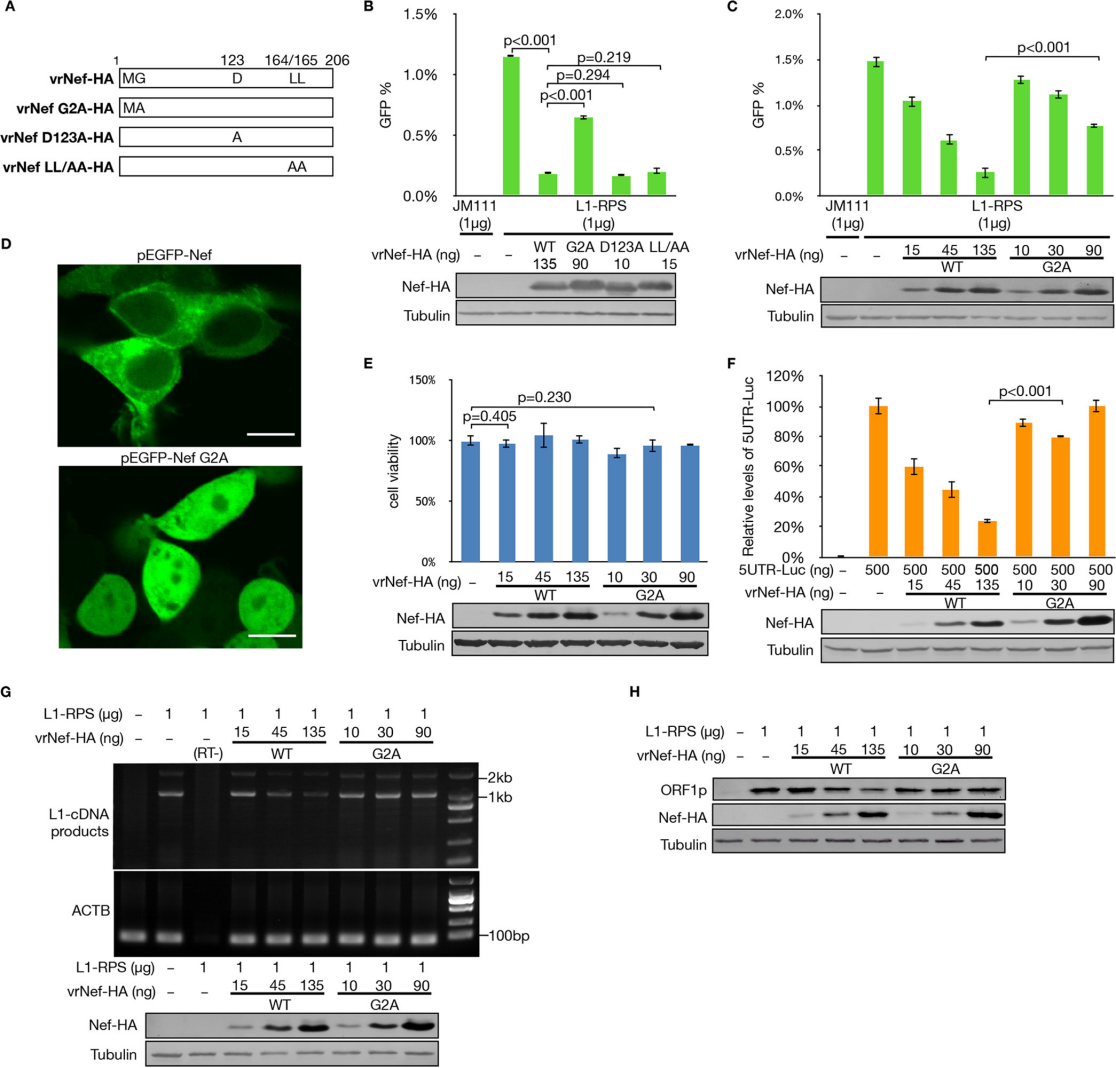
G2A mutation abolishes Nef’s ability to suppress the LINE-1 5′-UTR and compromises Nef-mediated LINE-1 suppression. (A) Schematics showing the Nef mutants used in this study. (B) Flow cytometry data showing that G2A, but not other mutations, significantly compromises Nef-mediated LINE-1 suppression. (C) Flow cytometry data confirming that, compared to wild-type Nef, Nef G2A in different dosages was less effective in LINE-1 inhibition. (D) Fluorescent images showing that the G2A mutation alters the subcellular localization of Nef in live HEK293T cells. EGFP was fused to the C terminus of the Nef protein and was thus used to indicate Nef distribution in the cells. Scale bars, 10 μM. (E) CCK-8-based cell viability test results indicating that neither wild-type Nef nor its G2A mutant could induce cytotoxicity in HEK293T cells. (F) Luciferase assay results indicating that Nef G2A no longer suppress the promoter activity of the LINE-1 5′-UTR. (G) Images of electrophoresis gel showing that, compared to wild-type Nef, Nef G2A does not downregulate levels of L1-RPS RNA. (H) Western blotting results showing that Nef G2A fails to reduce ORF1p expression from L1-RPS.

Nef is a multifunctional protein that downregulates the cell surface levels of CD4 and major histocompatibility class I (MHC-I) molecules, triggering cellular signaling and activation (44). It also promotes viral infectivity by reducing levels of SERINC proteins (40, 41). Several specific and essential residues on Nef are associated with these functions (Fig. 3A). Interestingly, among all the mutants tested, only Nef G2A showed weakened potency in LINE-1 suppression (Fig. 3B), which was further confirmed with both wild-type Nef and G2A expressed at different dosages (Fig. 3C). The glycine (G) residue at the second position of Nef is essential for myristoylation, which allows Nef to temporarily attach itself to the membrane structure inside the cell (45, 46). Fluorescence imaging of live HEK293T cells expressing EGFP-fused wild-type Nef or Nef G2A clearly indicated that the G2A mutation altered the cellular distribution of Nef (Fig. 3D). Thus, the ineffectiveness of Nef G2A in LINE-1 regulation indicated that subcellular localization is important for the inhibition of LINE-1 retrotransposition by Nef.

To further confirm that the G2A mutation indeed compromised the ability of Nef to suppress LINE-1, we tested Nef G2A for its efficacy against different LINE-1 components. First, cell viability tests based on CCK-8 suggested that neither wild-type Nef nor Nef G2A induced cytotoxicity in the HEK293T cells (Fig. 3E). It was then discovered that, unlike wild-type Nef, the G2A mutant could no longer regulate the promoter activity of the LINE-1 5′-UTR (Fig. 3F). Consequently, G2A failed to reduce the levels of LINE-1 RNA (Fig. 3G) or the protein levels of ORF1p (Fig. 3H). These results also indicated that Nef-induced 5′-UTR suppression contributes to Nef-mediated LINE-1 suppression.

### Nef indirectly compromises the interaction between ORF1p and LINE-1 RNA.

**FIG 4:**
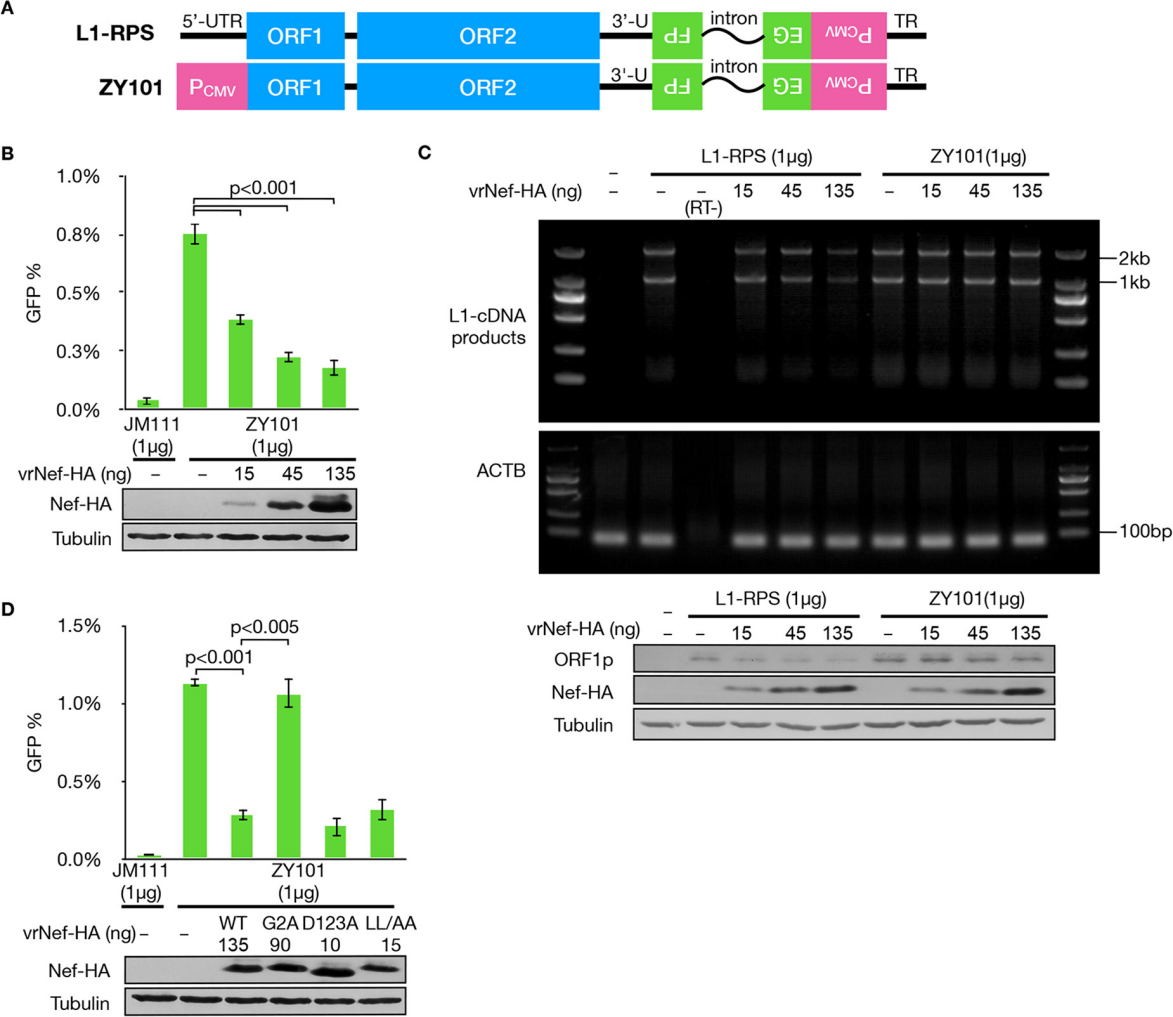
Nef regulates LINE-1 retrotransposition through a mechanism independent from 5′-UTR suppression. (A) Schematics showing differences between L1-RPS and ZY101; the latter has the LINE-1 5′-UTR fragment replaced with a CMV promoter. (B) Flow cytometry data suggesting that Nef potently suppresses the retrotransposition activity of ZY101. (C) Images of electrophoresis gel showing that Nef does not downregulate RNA levels transcribed from ZY101. (D) Flow cytometry data showing that Nef G2A is incapable of suppressing ZY101 retrotransposition, but other mutants are not.

To confirm that Nef suppresses LINE-1 replication through 5′-UTR regulation, we introduced an L1-RPS-based vector termed ZY101, which has the 5′-UTR replicated with a CMV promoter (47) (Fig. 4A). In theory, if 5′-UTR regulation is the sole mechanism for Nef-mediated suppression of LINE-1, Nef should not affect the retrotransposition efficiency of ZY101 because it has no impact on CMV promoter activity. However, when we performed a LINE-1 retrotransposition assay with ZY101, Nef was still able to suppress ZY101 (Fig. 4B). This suggests that an additional mechanism underlies Nef-mediated LINE-1 suppression. Consistent with the observation that Nef had no effect on CMV promoter activity (Fig. 1F and Fig. 2K), Nef did not reduce LINE-1 RNA levels transcribed from ZY101 or decrease the ORF1p levels expressed from ZY101 (Fig. 4C), indicating that this additional mechanism most likely affects LINE-1 activity at a posttranslational step. However, it is worth noting that the G2A mutation again compromised Nef’s potency against ZY101 retrotransposition (Fig. 4D), which provided an opportunity to elucidate the underlying mechanism.

**FIG 5:**
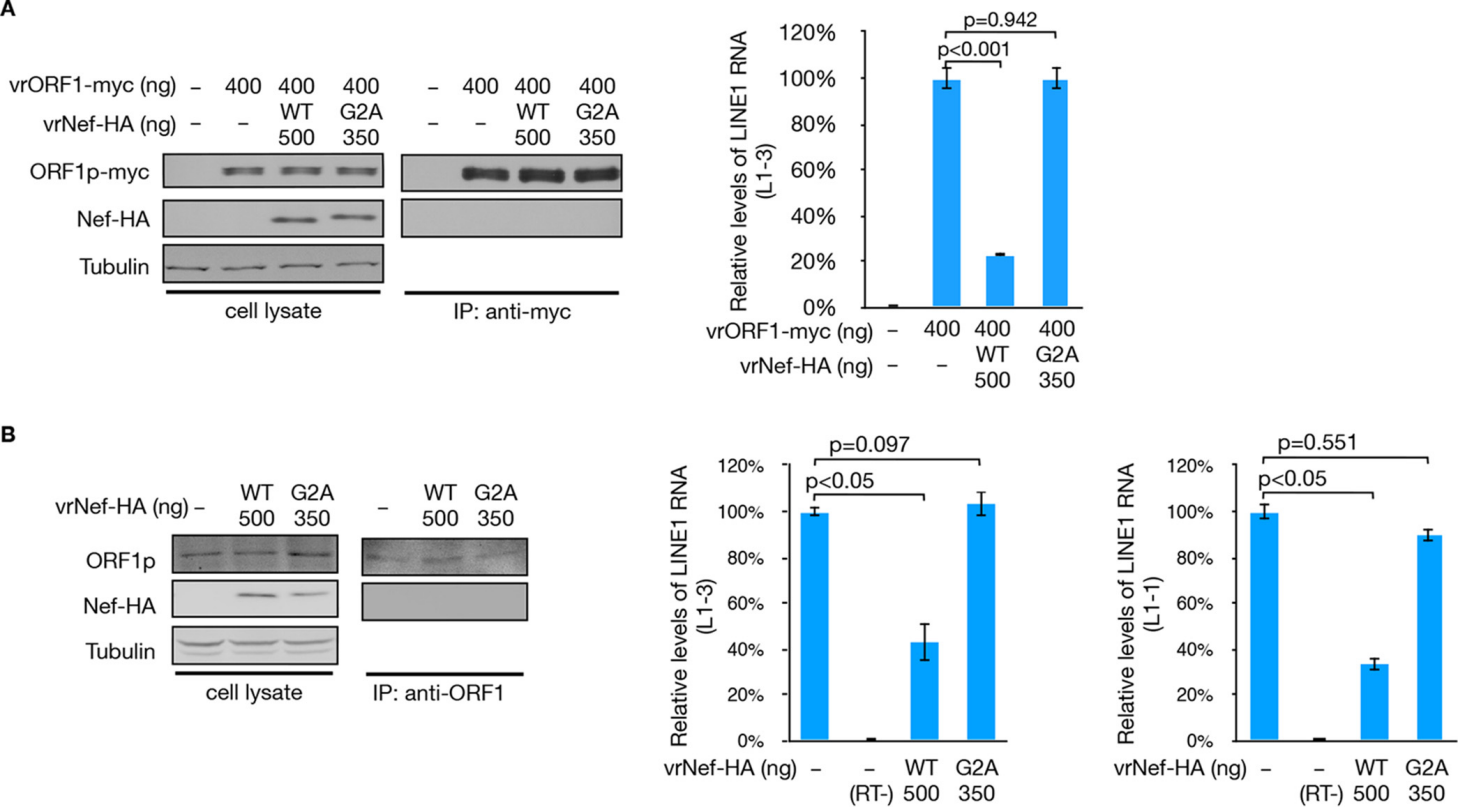
Nef compromises the interaction between ORF1p and LINE-1 RNA. (A) qRT-PCR results with the L1-3 primer pair suggesting that wild-type Nef, but not the G2A mutant, significantly compromises the interaction between exogenous ORF1p-myc and endogenous LINE-1 RNA in HEK293T cells. Western blotting results (left) show the extracted levels of ORF1p-myc in the co-IP experiment. (B) qRT-PCR results with both the L1-1 and L1-3 primer pairs suggesting that wild-type Nef, but not the G2A mutant, significantly compromises the interaction between endogenous ORF1p and endogenous LINE-1 RNA in HEK293T cells. Since no exogenous *orf1* fragment (DNA or RNA) was introduced, the L1-1 primer pair targeting *orf1* was also used to verify Nef’s interaction with ORF1p-LINE-1 RNA. Western blotting results (left) show the extracted levels of endogenous ORF1p in the co-IP experiment.

After translation, both ORF1p and ORF2p interact with their coding RNA and trigger the assembly of LINE-1 RNPs (48). We then checked whether Nef could compromise the integrity of LINE-1 RNPs by examining the interaction between ORF1p and LINE-1 RNA. To avoid Nef’s effect on the 5′-UTR and to focus on ORF1p, the vrORF1-myc vector containing a CMV promoter and expressing a myc-tagged ORF1p was used instead of a LINE-1-based construct (Fig. 2A). HEK293T cells were transfected with vrORF1-myc together with the vector expressing the wild-type Nef or Nef G2A mutant. The expressed ORF1p-myc proteins were then extracted through co-IP experiments, and the levels of bound LINE-1 RNA were examined through reverse transcription and qRT-PCR. The results confirmed that exogenous ORF1p protein interacted with endogenous LINE-1 RNA, which was significantly compromised in the presence of wild-type Nef (Fig. 5A). This was surprising because Nef did not interact with either ORF1p (Fig. 2E and Fig. 5A) or LINE-1 RNA (Fig. 2H). However, it is worth noting that Nef G2A, which barely inhibited ZY101 activity (Fig. 4D), did not affect the interaction between ORF1p and LINE-1 RNA (Fig. 5A). This suggests a correlation between the Nef-compromising ORF1p-LINE-1 RNA interaction and Nef-mediated suppression of LINE-1 retrotransposition. To further confirm this hypothesis and prevent any unexpected impact of the myc tag fused with ORF1p, we performed additional tests and targeted endogenous ORF1p in HEK293T cells. Similar to previous observations, wild-type Nef potently reduced the interaction between ORF1p and LINE-1 RNA, whereas Nef G2A did not (Fig. 5B). These findings demonstrate that Nef-induced disruption of ORF1p-LINE-1 RNA interaction contributes to Nef-mediated LINE-1 suppression.

### Nef suppresses LINE-1-induced innate immune activation.

**FIG 6:**
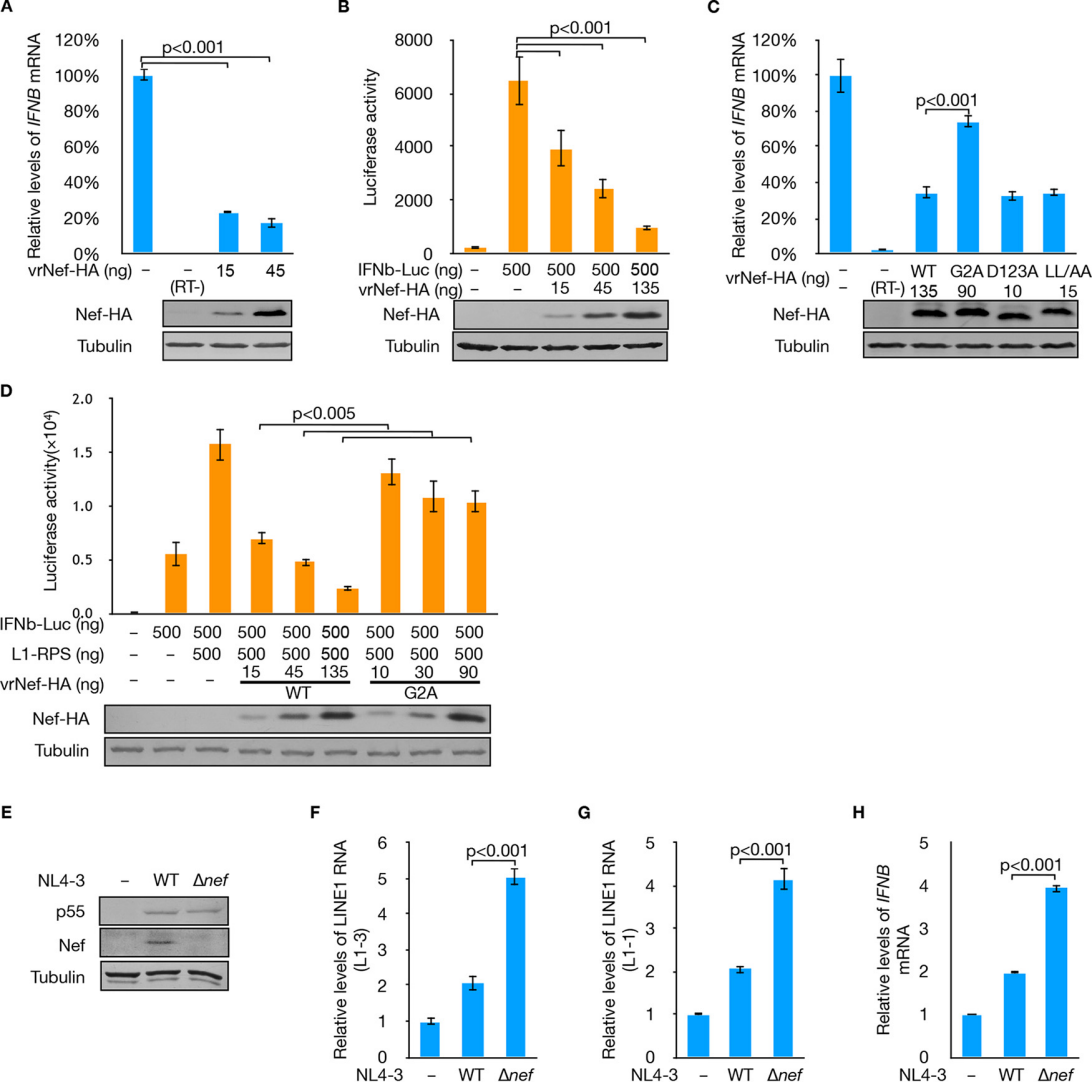
Nef reduces IFN production through LINE-1 suppression. (A) qRT-PCR results suggesting that Nef potently reduces the endogenous levels of *IFNB* mRNA. (B) Luciferase assay results indicating that Nef effectively suppresses *IFNB* promoter activity. (C) qRT-PCR results suggesting that G2A, but not other tested mutations, potently reduces Nef’s ability in *IFNB* mRNA regulation. (D) Luciferase assay results confirming that Nef G2A is much less effective in *IFNB* promoter suppression. (E to H) Nef expressed during HIV infection is potent in LINE-1 suppression and IFN regulation. Jurkat cells were infected with wild-type NL4-3 and NL4-3 Δ*nef* for 10 days. (E) Western blotting results confirm that similar infection rates were achieved for NL4-3 and NL4-3 Δ*nef* compared to protein levels of viral p55. (F to H) qRT-PCR results indicate that endogenous LINE-1 RNA and *IFNB* mRNA are significantly higher in NL4-3 Δ*nef*-infected Jurkat cells than those infected with wild-type NL4-3.

LINE-1 RNPs are the fundamental units of LINE-1 retrotransposition (48–50), as well as innate immune activation, acting as an endogenous trigger, as recently confirmed (25, 26, 32, 34). By suppressing LINE-1 transcription and translation and sabotaging the interaction between ORF1p and LINE-1 RNA, Nef theoretically reduces the formation of LINE-1 RNPs, Thus, through LINE-1 inhibition, Nef may regulate the activity of the innate immune system, where IFN production plays an important role. Indeed, we found that Nef reduced endogenous *IFNB* mRNA levels in HEK293T cells (Fig. 6A). Additional tests indicated that Nef suppressed endogenous IFN-β production by downregulating *IFNB* promoter activity (Fig. 6B). Notably, the G2A mutation, which compromised Nef’s potency in LINE-1 suppression, also affected Nef’s efficiency in IFN-β regulation (Fig. 6C). To further correlate the above phenomena with Nef-mediated inhibition of LINE-1, L1-RPS was transfected into HEK293T cells to trigger activation of the *IFNB* promoter (Fig. 6D). Wild-type Nef expressed at different dosages effectively reduced L1-RPS-triggered *IFNB* promoter activation, which was significantly compromised by the G2A mutation (Fig. 6D). Thus, Nef regulates IFN production via LINE-1 suppression.

These results were obtained in HEK293T cells expressing exogenous Nef. We subsequently determined whether Nef had a similar effect during viral infection. To this end, NL4-3 and NL4-3 Δ*nef* were used to infect Jurkat cells, which are T cell derivatives that are widely used for studying HIV infection *in vitro*. Similar infection rates were achieved for both wild-type NL4-3 and NL4-3 Δ*nef*, as shown by the expression levels of viral p55 protein (Fig. 6E). Interestingly, qRT-PCR targeting endogenous LINE-1 RNA suggested that NL4-3 infection mildly increased LINE-1 RNA levels in Jurkat cells (Fig. 6F and G), consistent with a previous study showing that NL4-3 enhances LINE-1 replication in the same type of cells (51). Nevertheless, compared to wild-type NL4-3, Jurkat cells infected with NL4-3 Δ*nef* contained higher levels of LINE-1 RNA (Fig. 6F and G). It has been reported that increasing the endogenous levels of LINE-1 RNA enhances IFN expression (26). Accordingly, *IFNB* mRNA levels were significantly higher in cells infected with NL4-3 Δ*nef* than in cells infected with wild-type NL4-3 (Fig. 6H). These data indicate that the viral Nef protein is potent in both LINE-1 inhibition and innate immune regulation during HIV infection.

## DISCUSSION

As a multifunctional protein, Nef has been found to have at least three *in vitro* activities, (i) Nef downregulates cellular surface proteins such as CD4 and major histocompatibility class I (MHC-I) molecules (52, 53); (ii) Nef mediates cellular signaling and activation (54); and (iii) Nef enhances viral infectivity by neutralizing restriction factors, such as SERINC5 and BST2 (40, 41, 55, 56). In this study, we found that Nef acts as an effective suppressor of LINE-1 retrotransposition. Nef from HIV NL4-3 demonstrated the potency of LINE-1 suppression in both LINE-1 retrotransposition assays based on EGFP detection and drug selection. Two distinct mechanisms have been identified for the Nef-mediated LINE-1 regulation. Nef suppressed the promoter activity of the LINE-1 5′-UTR, leading to decreased efficiency of LINE-1 RNA transcription and subsequent LINE-1 protein expression. In addition, although it does not directly interact with either component, Nef compromises the binding between ORF1p and LINE-1 RNA, thus compromising the formation/integrity of LINE-1 RNPs. Intriguingly, both mechanisms require the presence of the G2 residue in the Nef protein. Consistent with previous studies showing that LINE-1 activity triggers innate immune activation (25, 26, 34), Nef expressed from a eukaryotic expression vector or HIV proviral DNA was found to reduce IFN production, possibly through LINE-1 regulation.

Our findings indicate that the G2 residue is important for Nef-mediated suppression of LINE-1, which is not surprising since the same residue is essential for most functions of Nef, including the interaction and downregulation of CD4 (57) and the restriction of antiviral factors such as SERINC3 and SERINC5 (40, 41), as well as for enhancing HIV-1 infectivity (58). The G2A mutation used in this study has been reported to abolish myristoylation of Nef. Myristoylation grants Nef the ability to insert itself into the membrane structure (46). In other words, membrane localization appears to be critical for Nef-mediated LINE-1 suppression. Notably, both mechanisms act indirectly on LINE-1, although similar precedents have been observed for other LINE-1 suppressors. Indeed, Nef suppresses the promoter activity of the LINE-1 5′-UTR without directly interacting with LINE-1 DNA, which is similar to the previously reported mechanism of BST2-mediated LINE-1 suppression (32). In addition, Nef indirectly compromises ORF1p’s potency in binding LINE-1 RNA, whereas SAMHD1 was reported to relocalize ORF1p onto stress granules without direct interaction (59). However, it was surprising that one G2A mutation compromised both mechanisms of Nef-induced LINE-1 inhibition. One possibility is that Nef may interact with cellular factors that are critical for LINE-1 transcription and LINE-1 RNP stabilization, restricting these factors to membrane structures and preventing their interaction with LINE-1 components. Nevertheless, myristoylation appears to be essential for Nef-mediated LINE-1 suppression as well as Nef-induced IFN reduction.

It is well-known that IFN can significantly suppress HIV replication, mostly through its potency to trigger the expression of IFN-stimulated genes (ISGs), the products of which contain many restriction factors that are effective in viral inhibition (60). On the other hand, previous studies have indicated that HIV utilizes multiple mechanisms to reduce or evade innate immune activation to optimize its replication (19, 61–63). Therefore, Nef may enhance HIV replication by repressing IFN expression. Unfortunately, it is currently difficult to test the impact of Nef-mediated IFN regulation on HIV infection in systems such as THP-1 or even primary T cells that respond well to IFN stimulation. The key reason for such a setback is the G2 residue of Nef, which is not only critical for Nef-mediated LINE-1 suppression and IFN reduction but is also essential for most other functions of Nef, including Nef-induced depletion of anti-HIV factors. However, a previous study showed that in primary T cells where both anti-HIV SERINC3 and SERINC5 were knocked down, Nef still enhanced the infectivity of HIV (41), which increases the possibility that Nef-mediated IFN suppression contributes to HIV replication. Therefore, despite the need for designing a better system before examining the impact of Nef-mediated IFN regulation on HIV infection, our data (along with previously reported studies) demonstrate that Nef, with its multiple contributions to HIV pathogenesis, is a promising target for the design of new anti-HIV drugs.

Intriguingly, despite the identification of multiple viral proteins as potent LINE-1 suppressors, including Vpr (29), Vpu (33), and Nef (this study), HIV tends to maintain endogenous LINE-1 activity at a certain level. We noticed this because LINE-1 RNA was slightly elevated in Jurkat cells infected with wild-type NL4-3 (Fig. 6F and G). This is consistent with a previous study showing that HIV enhances LINE-1 retrotransposition in Jurkat cells or even primary T cells (51). One possibility is that being evolutionarily related to retroelements, modern retroviruses such as HIV may enhance retrotransposition due to similarities shared by the two. However, this will trigger IFN activation, which, in turn, will become an obstacle for HIV replication; thus, HIV has evolved additional mechanisms to suppress retrotransposition. Another reason for such a phenomenon is that although the high retrotransposition rate of LINE-1 triggers innate immune activation that would suppress the infectivity of HIV, certain steps of LINE-1 (e.g., genome nicking) by themselves may be important for HIV replication. For instance, LINE-1 can arrest the cell cycle at the G_2_/M phase by inducing genome damage (13, 24), whereas HIV replication has been reported to be more effective in cells arrested at the G_2_/M phase (28, 64). As a result, instead of suppressing LINE-1 retrotransposition and LINE-1-induced IFN production in a more sufficient way, HIV chooses to maintain certain levels of LINE-1 activity to optimize viral replication. Apparently, as we previously predicted (65), the relationship between HIV, LINE-1, and the IFN signaling system is intricate, and further details are yet to be revealed.

## MATERIALS AND METHODS

### Cells and plasmids.

HEK293T and HeLa cells were cultured in Dulbecco’s modified Eagle’s medium (DMEM; catalog no. C11995500BT; Gibco), while Jurkat cells were cultured in RPMI 1640 (catalog no. C11875500BT; Gibco). Both media were supplemented with 10% fetal bovine serum (FBS; catalog no. 04-001-1ACS; Biological Industries) and penicillin-streptomycin (Pen-Strep; catalog no. 03-031-1B; Biological Industries).

The retrotransposition-competent vector 99 PUR RPS EGFP (L1-RPS) (37), the retrotransposition incompetent 99 PUR JM111 EGFP (JM111) (37), LcRPS-mneoI (L1-neo), pJM111 (JM111-neo) (66), pc-L1-RPS-Δ5UTR(ZY101) (47), VR1012 (35), VR1012-ORF1-Myc (vrORF1-myc) (20), VR1012-ORF1-HA (vrORF1-HA) (32), pGL3-IFNB-Luciferase (IFNB-Luc) (67), pGL3-5′-UTR-Luciferase (5UTR-Luc) (20), pGL3-CMV-Luciferase (CMV-Luc) (20), and the HIV proviral DNA vector pNL4-3 have been described previously. To generate vrNef-HA, the *nef* gene was amplified from pNL4-3 using a stranded PCR and introduced into VR1012. Point mutations were introduced into vrNef-HA using stranded point-directed mutagenesis. To generate vrORF1 expressing untagged ORF1p, the *orf1* fragment was retrieved from vrORF1-myc and inserted into VR1012. In addition, the *trex1* gene was amplified from the previously reported vrTREX1-Flag vector (20) and inserted into VR1012 to generate vrTREX1-HA.

To generate pNL4-3 Δ*nef*, a stop codon was introduced next to the start codon of the *nef* gene in the pNL4-3 vector through site-directed mutagenesis. The expression levels of Nef in pNL4-3 Δ*nef* were verified by Western blotting.

Transfection assays were performed using Lipofectamine 3000 reagent (catalog no. L3000015; Invitrogen) and Opti-MEM (catalog no. 802679; Gibco) according to the manufacturers’ protocols.

### Antibodies and reagents.

The following antibodies were used in this study: anti-tubulin (catalog no. HC101-02; TransGen), anti-HA (catalog no. 901513; BioLegend), anti-Myc tag antibody, clone 4A6 (catalog no. 05-724; Millipore), anti-LINE-1 ORF1p, clone 4H1 (catalog no. MABC1152; Millipore), anti-Nef (catalog no. ab42358; Abcam), and anti-p24 (catalog no. 1513; AIDS Research and Reference Reagents Program). All antibodies were used according to the manufacturers’ protocols. Puromycin was purchased from Sigma (product no. P8833; 25 mg), and G418 was purchased from TCI (catalog no. TCI-G0349).

### LINE-1 retrotransposition assay.

The LINE-1 retrotransposition assay was performed as previously described (17, 37). L1-RPS is based on L1_RP_, with an antisense EGFP reporter cassette in the 3′ UTR, whereas the *EGFP* gene is interrupted by a sense group I intron. EGFP can only be detected when the LINE-1 transcript is spliced and reverse transcribed, its cDNA is inserted into the host genome, and the *EGFP* reporter gene is expressed by its own CMV promoter. JM111 is similar to L1-RPS, which contains two point mutations in ORF1p that completely abolish retrotransposition. Briefly, 1 μg of L1-RPS or JM111 was transfected into HEK293T cells in 24-well plates with VR1012 or one of the test plasmids. At 48 h posttransfection, the cells were subjected to selection with 5 μg/mL puromycin and were tested by flow cytometry using FACSCalibur after another 48 h. Gating exclusion was based on the background fluorescence of plasmid JM111. A total of 20,000 single-cell events per sample were collected and analyzed using FlowJo software (version 7.6.1).

To confirm that the observed effects acted on LINE-1, another LINE-1 retrotransposition assay based on neomycin resistance was performed. The plasmids L1-neo and JM111-neo were similar to L1-RPS and JM111, respectively, with the antisense CMV-EGFP cassette changing into an SV40-mneoI cassette. Accordingly, cell clones that emerged under neomycin (G418) selection were considered to be positive signals for LINE-1 retrotransposition. Notably, JM111-neo is based on L1.2 instead of L1_RP_ (38). It also has a CMV promoter at the 5′ end of the LINE-1 sequence. These different features did not affect the use of JM111-neo, though, as JM111-neo was used as the negative control and barely generated positive clones. Briefly, 1 μg of L1-neo or JM111-neo was transfected into HeLa cells in 24-well plates with VR1012 or one of the test plasmids. At 48 h posttransfection, the cells were transferred to a 6-well plate at a ratio of 1:20 and treated with 400 μg/mL G418 for 9 days. The cells were fixed with phosphate-buffered saline (PBS), paraformaldehyde, and glutaraldehyde, and the colonies were stained with 0.4% Giemsa.

### Quantitative real-time reverse transcription-PCR.

Total RNA from samples of interest was extracted using the FastPure cell/tissue total RNA isolation kit (catalog no. RC101; containing DNase treatment; Vazyme) and subjected to reverse transcription with MonScipt RTIII All-in-One mix (catalog no. RN05004M, including DNase treatment before reverse transcription; Monad). An RT^−^ control (without reverse transcriptase) was prepared for each test and used as a parallel sample to detect any possible contamination of genomic DNA (data not shown). qRT-PCR experiments were performed using the MonAmp ChemoHS qPCR mix (catalog no. RN04001N; Monad) and specific primers. The reactions were performed according to the manufacturer’s instructions as follows: 94°C for 30 s, followed by 40 cycles of 94°C for 10 s and 60°C for 30 s, followed by a dissociation protocol. Single peaks in the melting curve analysis indicated the presence of specific amplicons. *ACTB* mRNA was used as a cellular mRNA control (data not shown). The primers used were L1-1, forward (5′-GAATGATTTTGACGAGCTGAGAGAA-3′) and reverse (5′-GTCCTCCCGTAGCTCAGAGTAATT-3′); L1-3, forward (5′-CAAACACCGCATATTCTCACTCA-3′) and reverse (5′-CTTCCTGTGTCCATGTGATCTCA-3′); 5UTR-1, forward (5′-GTGAGCGACGCAGAAGACG-3′) and reverse (5′-GGTGGGAGTGACCCGATTT-3′); 5UTR-2, forward (5′-GTGAGCGACGCAGAAGACG-3′) and reverse (5′-TAAGCAAGCCTGGGCAATG-3′); *IFNB*, forward (5′-AGGACAGGATGAACTTTGAC-3′) and reverse (5′-TGATAGACATTAGCCAGGAG-3′); and *ACTB*, forward (5′-ACCGAGCGCGGCTACAG-3′) and reverse (5′-CTTAATGTCACGCACGATTTCC-3′).

### PCR assay.

To determine the effect of Nef on full-length LINE-1 RNA, a previously reported PCR assay was performed (20). Briefly, 1 μg of L1-RPS and Nef-expressing vectors (15, 45, and 135 ng) were cotransfected into HEK293T cells. The cells were then subjected to RNA extraction and reverse transcription at 48 h posttransfection. PCR was performed on the synthesized cDNA using a 2× Phanta Max master mix (catalog no. P515; Vazyme). The reactions were performed according to the manufacturer’s instructions as follows: 95°C for 3 min, followed by 30 cycles of 95°C for 15 s, 56°C for 15 s, 72°C for 90 s, and 72°C for 5 min.

The levels of *ACTB* mRNA (used as a cellular mRNA control) were monitored using the above-described primer pairs. The levels of L1-RPS RNA were detected using L1-3 forward and *EGFP*-1 forward (5′-TGACCCTGAAGTTCATCTGC-3′). The amplicon covered regions from both the LINE-1 fragment and EGFP cassettes. The antisense EGFP cassette in L1-RPS had its own poly(A) signal; thus, the transcription of *EGFP* mRNA did not contain a LINE-1 fragment. Meanwhile, the *EGFP* gene does not exist in the human genome; therefore, the LINE-1 part ensured that the amplicon represented LINE-1 RNA transcribed from the 5′-UTR of L1-RPS instead of the antisense CMV promoter, whereas the EGFP part prevented possible contamination from endogenous LINE-1 DNA/RNA. An RT^−^ control for each sample was also included to eliminate possible contamination with L1-RPS DNA. Thus, the amplicons based on L1-3 forward and *EGFP*-1 forward represented full-length LINE-1 RNA transcribed from L1-RPS.

### Coimmunoprecipitation.

Co-IP experiments were performed as previously described (68). To confirm whether Nef interacts with ORF1p, HEK293T cells were transfected with TREX1 (600 ng), Nef (500 ng), and/or ORF1p-expressing vectors (400 ng) and then harvested at 48 h posttransfection. The samples were washed with 1× PBS, suspended in lysis buffer (50 mM Tris-HCl [pH 7.5], 150 mM NaCl, and 0.5% NP-40, supplemented with cOmplete tablets EDTA free, EASYpack; Roche), sonicated at 15% power for 15× 3-s breaks separated at 3-s intervals, and centrifuged at 12,000 rpm for 10 min to harvest the supernatant. The input samples were incubated with anti-HA magnetic beads (catalog no. 88837; Thermo Pierce) overnight and then washed six times with wash buffer (20 mM Tris-HCl [pH 7.5], 100 mM NaCl, 0.1 mM ethylenediaminetetraacetic acid [EDTA], and 0.05% Tween 20). The samples were then eluted with 50 mM glycine-HCl (pH 2.5) and subjected to subsequent analyses.

To determine whether Nef affected the interaction between ORF1p and LINE-1 RNA, 400 ng of vrORF1-myc and 500 ng of vrNef-HA were cotransfected into HEK293T cells, harvested at 48 h posttransfection, and subjected to co-IP experiments, where anti-myc beads (catalog no. 88842; Thermo Pierce) were used to extract ORF1p-myc and its interactants (or protein G saturated with anti-ORF1p antibody to extract endogenous ORF1p). The eluted samples were subjected to RNA extraction, reverse transcription, and qRT-PCR to confirm the levels of LINE-1 RNA.

### Luciferase reporter assay.

The TransDetect single-luciferase reporter assay kit (FR101-01; TransGen) was used to determine whether Nef affected the promoter activity of the LINE-1 5′-UTR and the *IFNB* promoter. Briefly, vrNef-HA (15, 45, 135 ng) and 500 ng of 5UTR-Luc or IFNB-Luc were cotransfected into HEK293T cells. At 48 h posttransfection, luciferase activity was measured according to the manufacturer’s protocol. Measurements of pGL3-transfected sample were used to remove background noise and are not shown.

### Live-cell fluorescence imaging.

To confirm that the G2A mutation altered the subcellular localization of Nef, the *nef* gene was inserted into pEGFP-N1 to generate pEGFP-Nef, which expressed wild-type Nef with its C terminus fused with EGFP. A similar strategy was applied to generate pEGFP-Nef G2A.

HEK293T cells seeded on a 35-mm glass-bottom dish were then transfected with 600 ng of pEGFP-Nef or pEGFP-Nef G2A by using Lipofectamine 3000. At 24 h posttransfection, the cells were subjected to confocal imaging for EGFP signals by using a Zeiss LZM710 confocal microscope.

### HIV production and infection.

HEK293T cells seeded on a 6-cm plate were transfected with 8 μg of pNL4-3 or pNL4-3 Δ*nef* by using Lipofectamine 3000. The medium was changed 24 h posttransfection, and the supernatant was collected after an additional 24 h. After the cells and debris were removed by filtration, the virus titers were standardized with Testing Kit for HIV p24 Antigen (enzyme-linked immunosorbent assay [ELISA]) used only *in vitro* (Biomedical Engineering Center, Hebei Medical University). For viral infection, equal amounts of viruses were used to infect Jurkat cells seeded on 6-cm plates. The cells were collected 10 days postinfection and subjected to subsequent analyses.

### Quantification and statistical analysis.

Flow cytometry data are presented as the means ± standard deviations (SDs) of three technical replicates per experiment. Other data are presented as means ± standard errors of the mean (SEMs) of at least three independent biological replicates. ImageJ software was used to quantify relative levels of proteins in Western blotting results. Data were analyzed using unpaired two-tailed Student’s *t* tests, performed using Microsoft Excel.
